# Epidemiological tracing of *Batrachochytrium salamandrivorans* identifies widespread infection and associated mortalities in private amphibian collections

**DOI:** 10.1038/s41598-018-31800-z

**Published:** 2018-09-14

**Authors:** Liam D. Fitzpatrick, Frank Pasmans, An Martel, Andrew A. Cunningham

**Affiliations:** 1 Institute of Zoology, Zoological Society of London, Regent’s Park, London, NW1 4RY UK; 2 Department of Pathology, Bacteriology and Avian Diseases, Faculty of Veterinary Medicine, Ghent University, Salisburylaan 133, 9820 Merelbeke, Belgium

## Abstract

The amphibian chytrid fungus *Batrachochytrium salamandrivorans* (*Bsal*) infects newts and salamanders (urodele amphibians), in which it can cause fatal disease. This pathogen has caused dramatic fire salamander population declines in Belgium, the Netherlands and Germany since its discovery in 2010. Thought to be native to Asia, it has been hypothesised that *Bsal* was introduced to Europe with the importation of infected amphibians for the commercial pet trade. Following the discovery of *Bsal* in captive amphibians in the United Kingdom in 2015, we used contact-tracing to identify epidemiologically-linked private amphibian collections in Western Europe. Of 16 linked collections identified, animals were tested from 11 and urodeles tested positive for *Bsal* in seven, including the identification of the pathogen in Spain for the first time. Mortality of *Bsal*-positive individuals was observed in five collections. Our results indicate that *Bsal* is likely widespread within the private amphibian trade, at least in Europe. These findings are important for informing policy regarding *Bsal* control strategies.

## Introduction

Emerging infectious diseases pose a substantial threat to global biodiversity, causing population declines, and even species extinctions, across a range of taxa^1^. The amphibian chytrid fungus *Batrachochytrium salamandrivorans* (*Bsal*) is a current example of this, being the putative cause of an over 99% decline in a monitored population of fire salamanders (*Salamandra salamandra*) in the Netherlands^2^. The pathogen and consequent fire salamander population declines have since expanded into Belgium and Germany^3^. Experimental infection trials and field observations indicate that, although *Bsal* can also infect anuran amphibians^4,5^, it is only known to cause disease in amphibians of the order Urodela (newts and salamanders)^6^. This is unlike its sister fungus, *Batrachochytrium dendrobatidis* (*Bd*), which can infect and cause disease in amphibians across all three amphibian orders (Anura, Urodela and Gymnophiona)^7–9^.

Infection experiments demonstrated that urodeles native to Europe were either killed by, or were resistant to, *Bsal* infection; however some Asian urodeles were shown to survive infection without mortality^6^. In three Asian species tested (*Cynops cyanurus, Cynops orientalis* and *Paramesotriton deloustali*), all individuals developed clinical disease, but some cleared infection without treatment or maintained infection for an extended period of time^6^. One Asian urodele species, *Salamandrella keyserlingii*, was shown to tolerate infection in the absence of disease^6^. Most of the experimentally-infected animals were captive-bred (animals for only five species tested were wild-caught)^6^, a factor which might influence infection outcome. These infection trials, however, provide the best available data on the response of different urodele species to exposure to *Bsal*.

These findings, in combination with a global surveillance effort of wild salamanders that identified *Bsal* only in the Netherlands, where epidemic disease was occurring, and in Asia (Thailand, Vietnam and Japan), led to the hypothesis that the fungus is endemic to Asia^4^. The subsequent identification of widespread *Bsal* infection, in the absence of disease, in wild urodeles in Vietnam and China^10,11^, supports the hypothesis that urodeles from this region are reservoir hosts of the pathogen. Until recently, wild-caught urodeles from Asia were routinely imported into Europe as part of the pet trade^12^, and this is considered to have been the likeliest route for the introduction of *Bsal* into Europe^6^. Such introduction of non-native pathogens into new geographic regions with naive host species/populations is a recognised driver of infectious disease emergence and has been termed “pathogen pollution”^13^.

Despite difficulties in tracking and quantifying the global amphibian trade^14^, Europe is known to be a major importer of live amphibians^15^. In the United Kingdom (UK) alone, an estimated 131,000 live amphibians were imported in 2006, with approximately 98% believed to be for the pet trade^16^. Amphibian trade, for purposes such as research, food and the pet trade, has already been implicated in the global spread of *Bd*^14,17^, which has led to declines or extinctions of hundreds of amphibian species^18,19^. Thus, analysing the current state of *Bsal* infection in the amphibian trade is essential to understand the risk of further *Bsal* incursions into wild populations.

The private amphibian trade (*i.e*. the movement of animals between individual collectors who breed and sell amphibians on a non-commercial scale) takes place across Western Europe. Such trade is often directly between collectors or at large-scale fairs^20^, with little legislation governing the practice^15^. In the absence of sanitary regulations or practices, this trade presents a potential route of pathogen movement across international borders, particularly in animals which carry pathogens without any obvious signs of disease^20^. Reports of *Bsal* chytridiomycosis outbreaks in captive amphibian collections in the UK and Germany have shown that *Bsal* can cause mortality in captive animals^21,22^. The initial detection of *Bsal* in the UK was in 2015, in animals recently obtained by a zoological collection from a private amphibian breeder also in the UK^21^. This current study follows on from that initial detection, where using contact-tracing methods, we identified epidemiologically-linked amphibian collections across Western Europe and, where possible, we sampled urodeles in these collections to test for the presence of *Bsal*.

## Results

### Epidemiological tracing

**Figure 1:**
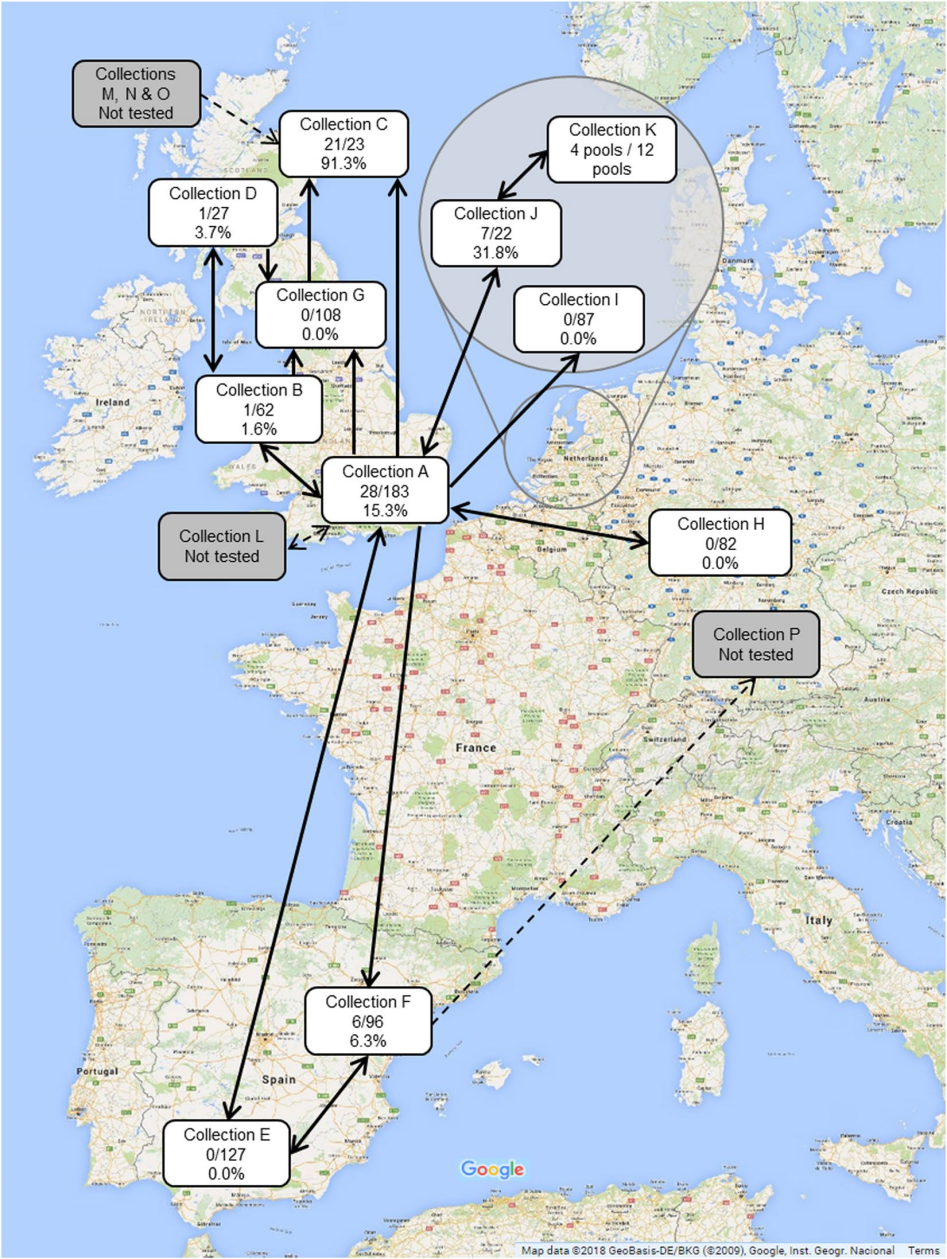
Epidemiological links between all sixteen contact-traced private collections. Access was granted to Collections (A-K) with all urodeles swabbed for *Bsal* infection. Access was not granted to Collections (L-P). Arrows indicate the direction of transit of animals, including those subsequently identified as *Bsal*-positive. Numbers and percentages indicate the number of *Bsal*-positive/total number of urodeles tested.

Sixteen private amphibian collections were identified as being epidemiologically linked to the index (first) case of *Bsal* infection in the UK^21^. We identified Collection A as the source of the *Bsal* infected urodeles in the index zoological collection^21^. Further epidemiological links between private collections were established either through the sale, purchase or swapping of urodeles during the previous 24 months (Fig. 1). Of the 16 linked collections, 11 collectors granted access to sample amphibians for *Bsal* infection (Collections A-K), and five collectors denied access (Collections L-P). Summary information on Collections A-K, including the total numbers of urodeles and numbers of species held at the time of testing, is provided in Table 1. Access to each collection was allowed on the condition of anonymity. As details of the actual numbers of each species held in each location could identify some of the collections, these data have not been shown, but a list of the total number of each species examined is listed in Supplementary Tables S1 and S2.

**Table 1:** Location, number of individual urodeles, number of species sampled and the number and proportion of *Bsal*-positive animals for each private amphibian collection at the time of testing.

Collection	Country	N^o.^ individual urodeles sampled	N^o.^ *Bsal*-positive animals	N^o.^ urodele species sampled	*Bsal* prevalence (95% CI – Clopper-Pearson)	Higher than usual number of mortalities reported?	Asian urodeles held in collection?
Collection A	United Kingdom	183	28	25	0.1530 (0.1041–0.2135)	Yes	No
Collection B	United Kingdom	62	1	10	0.0161 (0.0004–0.0866)	No	No
Collection C	United Kingdom	23	21	6	0.9130 (0.7196–0.9893)	Yes	No
Collection D	United Kingdom	27	1	7	0.0370 (0.0009–0.1897)	No	Yes
Collection E	Spain	127	0	15	0.0000 (0.0000–0.0286)	No	Yes
Collection F	Spain	96	6	22	0.0625 (0.0233–0.1311)	Yes	Yes
Collection G	United Kingdom	108	0	11	0.0000 (0.0000–0.0333)	No	Yes
Collection H	Germany	82	0	6	0.0000 (0.0000–0.0440)	No	No
Collection I	The Netherlands	87	0	8	0.0000 (0.0000–0.0415)	No	Yes
Collection J	The Netherlands	22	7	6	0.3182 (0.1386–0.5487)	Yes	No
Collection K	The Netherlands	60	4 groups	12	N/A	Yes	Yes

Also, whether or not higher than usual mortalities were reported by the collector at the time of swabbing and whether the collection held Asian urodeles are indicated. Each animal was sampled using a separate swab apart from animals in Collection K, where each swab was used to sample five animals; a total of 12 groups of five urodeles were sampled in this way.

Collectors reported mostly trading urodeles in person, meeting either privately or at large-scale fairs, where both commercial breeders and private collectors bring urodeles to trade with other collectors. Some collectors reported sending or receiving urodeles via postal and courier services. Trading often occurred in small numbers (less than ten urodeles), primarily to provide collections with a new species or to supplement currently held animals and to form breeding pairs. Trading at large-scale fairs was reported as common. Private hobbyist trade deals were often pre-agreed using social media, with collectors advertising which species they had available for sale or swap, and those species they wished to buy or swap in return.

### *Bsal* prevalence in private collections

At least one urodele tested positive for *Bsal* using qPCR in seven of the 11 tested collections: four in the UK, two in the Netherlands and one in Spain. Infection prevalences ranged from 1.6% (1/62) to 91.3% (21/23) across the collections at the time of testing (Table 1; Fig. 1). In accordance with the owner’s wishes, samples from live urodeles in Collection K were grouped by species (five animals per swab), so the prevalence of infection in this collection could not be ascertained. All 84 *Bsal*-positive urodeles across all collections were adults, 74 of which were captive-bred and ten were wild-caught. None of the 58 anurans of 21 species sampled in Collection A tested positive for *Bsal* using qPCR (Supplementary Table S2).

A comparison of *Bsal* prevalence in Asian (0/79) versus non-Asian (64/738) urodeles in Collections A-J showed a statistically significant difference (χ^2^ = 5.6651, p = 0.01731) with non-Asian urodeles having a higher prevalence of *Bsal* infection (Supplementary Table S1). Fisher’s exact tests showed no association between the presence of Asian species in a collection and higher than usual levels of mortality (p = 0.5671), or between the presence of Asian species and the presence of *Bsal* in a collection (p = 0.5455) (Table 1).

### Pathological examinations

Unusually high mortalities were reported from five of the tested collections (A, C, F, J and K; Table 1), all of which had mortalities associated with *Bsal*-positive urodeles.

The owner of Collection A donated 11 dead urodeles and two sick *Lissotriton boscai* (which were then euthanased) for pathological examination. No gross skin lesions were observed in any of the dead urodeles examined, however cachexia and ataxia, two non-specific signs associated with *Bsal* chytridiomycosis^2^, were observed in both *L. boscai* prior to euthanasia. Eleven of the 13 donated urodeles from Collection A tested positive for *Bsal* on qPCR, with infection intensities available for ten ranging from 1.2 to 2,836.92 genome equivalents (GE) swab^−1^ (Table 2). Of these, six (2 *Salamandra corsica*, 2 *S. salamandra* and both euthanased *L. boscai*) were suitable for post-mortem examination. All six necropsied animals were in a thin or emaciated body condition, with reduction or complete absence of coelomic fat bodies.

**Table 2:** *Bsal* infection intensities from urodeles that were euthanased or found dead.

Species	Collection	Confirmed *Bsal* chytridiomycosis on histopathology	Infection intensity (GE swab^−1^)
*Lissotriton boscai* ^*^	Collection A	Yes	523.20
*Lissotriton boscai* ^*^	Collection A	Not examined	2,836.92
*Lissotriton boscai*	Collection A	Not examined	108.24
*Salamandra atra*	Collection A	Not examined	Not done
*Salamandra atra*	Collection A	Not examined	1.2
*Salamandra corsica*	Collection A	No	2.16
*Salamandra corsica*	Collection A	No	42.12
*Salamandra salamandra*	Collection A	Not examined	1,720.32
*Salamandra salamandra*	Collection A	Not examined	69.84
*Salamandra salamandra*	Collection A	Not examined	43.32
*Salamandra salamandra*	Collection A	No	2.88
*Salamandra salamandra*	Collection C	Not examined	8,402.64
*Triturus marmoratus*	Collection F	Not examined	8,045.28
*Triturus marmoratus*	Collection F	Not examined	9,259.80
*Neurergus strauchii*	Collection J	Yes	Not done
*Neurergus strauchii*	Collection J	Yes	Not done
*Neurergus strauchii*	Collection J	Yes	Not done
*Notophthalmus viridescens*	Collection J	Yes	Not done
*Notophthalmus viridescens*	Collection J	Yes	Not done
*Notophthalmus viridescens*	Collection J	Yes	Not done
*Triturus macedonicus*	Collection K	Yes	40,900.00
*Triturus macedonicus*	Collection K	Yes	115,400.00

^*^Euthanased animals.

*Bsal* chytridiomycosis was confirmed when lesions consistent with the disease were seen on histopathological examination and when the animal was positive for *Bsal* using qPCR.

**Figure 2:**
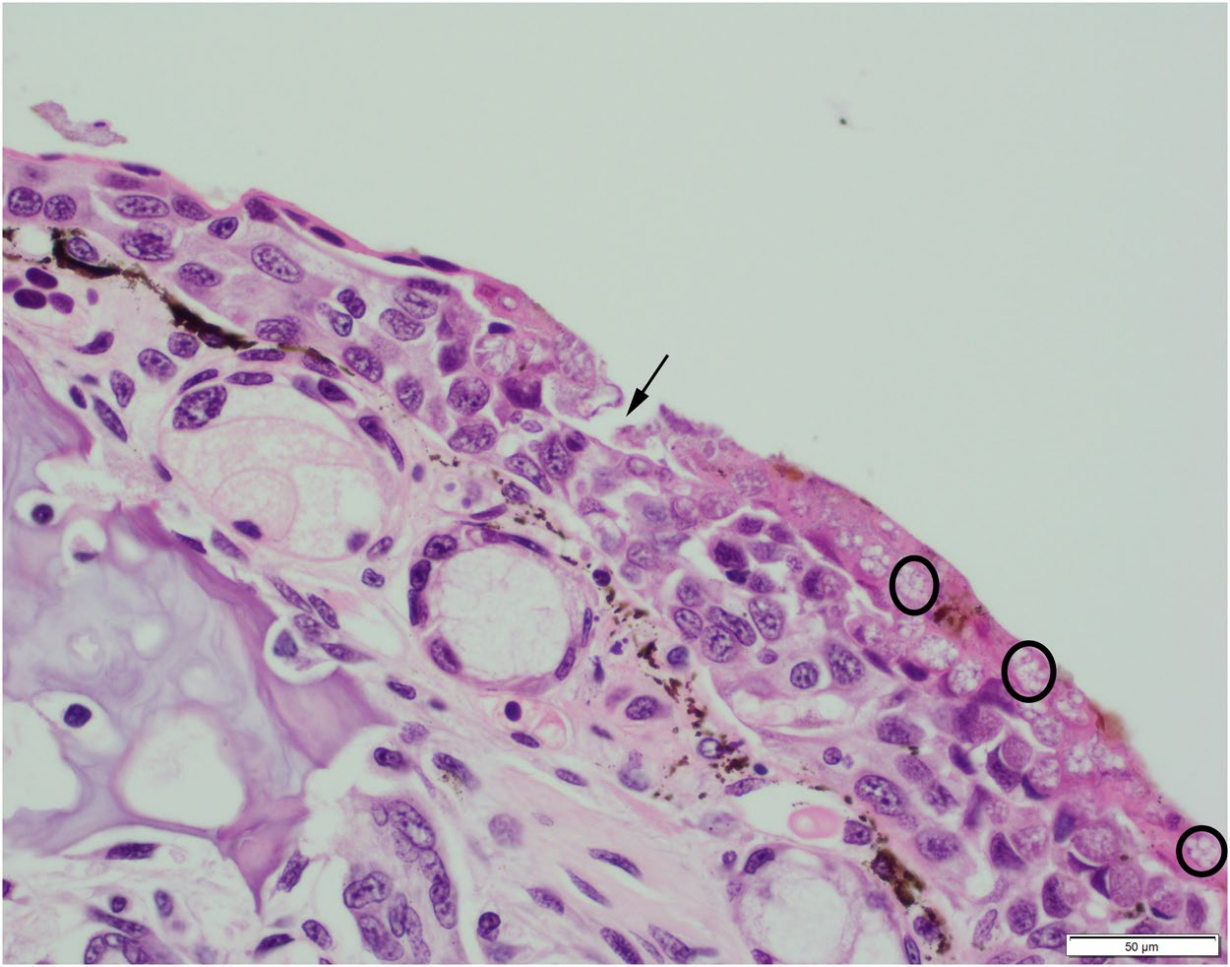
Section of hindlimb digit from a *Lissotriton boscai* euthanased on welfare grounds from Collection A (stained with haematoxylin and eosin). Black arrow indicates an epidermal erosion. Fungal zoosporangia (circled) are visible throughout the epidermis.

Samples were taken for histological examination from one *L. boscai*, one *S. salamandra* and two *S. corsica*. On histological examination of a forelimb and a hindlimb from the *L. boscai*, multiple areas of epidermal erosion associated with intracellular zoosporangia consistent with those of a chytrid fungus were identified (Fig. 2). *Bsal* chytridiomycosis was confirmed in this individual (Table 2), which had been captive-bred in Collection E and traded to Collection A in March 2013. No lesions consistent with *Bsal* chytridiomycosis were identified on histological examination of samples taken from the other three animals.

One *S. salamandra* was found dead in its enclosure in Collection C at the time of swabbing. This animal had a *Bsal* infection intensity of 8,402.64 GE swab^−1^. The two remaining live *S. salamandra* in this enclosure also tested positive for *Bsal* on qPCR; both had higher infection intensities than the dead individual (9,752.76 and 45,250.68 GE swab^−1^). The individual with the higher infection intensity was reported dead approximately one week later; the outcome of the other is unknown. The owner of Collection C also reported that four other known *Bsal-*positive animals, three *Lissotriton helveticus* and one *Triturus cristatus*, died approximately one week after swabbing. However, as these animals were not individually identifiable and were in an enclosure with conspecifics, we are unable to determine their infection intensities at the time of swabbing. Although these six dead urodeles were *Bsal*-positive, none were made available for pathological examination, so chytridiomycosis could not be confirmed as the cause of death.

In Collection F, two *Triturus marmoratus* were found dead in their enclosure at the time of swabbing. Four apparently-healthy individuals of the same species shared the enclosure. The two dead animals had substantially higher infection intensities (8,045.28 and 9,259.80 GE swab^−1^) (Table 2) than the four that remained alive (146.16, 348.60, 382.80 and 1,132.32 GE swab^−1^), but these four animals were reported to have died approximately two weeks after the swabbing took place. None of the six *T. marmoratus* were made available for pathological examination, so chytridiomycosis could not be confirmed as the cause of death.

Collection J experienced the complete loss of 160 animals of nine species from the genera *Lissotriton, Neurergus, Notophthalmus* and *Triturus* over the course of 2015. The collector submitted six captive-bred urodeles (three *Neurergus strauchii* and three *Notophthalmus viridescens*) that died during this period for pathological examination. All six tested positive for *Bsal* on qPCR, but the infection intensities are not available, and all had multiple skin erosions observed on histological examination. *Bsal* chytridiomycosis was confirmed in all six cases (Table 2).

Collection K reported the complete loss of 12 urodelan breeding groups in a period of three months, including fully-aquatic newts of species from the genera *Euproctus, Neurergus, Ommatotriton* and *Triturus*. The collector submitted two *Triturus macedonicus* that died during these mortality events for pathological examination. Both *T. macedonicus* were captive-bred and were in the aquatic breeding stage at the time of death. *Bsal* chytridiomycosis was confirmed in both cases using a combination of histopathological examination and qPCR (Table 2)^23^. Skin swabs taken from the two dead *T. macedonicus* had infection intensities of 40,900.00 and 115,400.00 GE swab^−1^.

### *Bsal* genotyping

Sequencing of amplicons obtained following PCR^2^ of DNA extracted from skin-swabs from three *Bsal*-positive *S. salamandra* from Collection A and from *Bsal* isolates cultured from one diseased *Neurergus strauchii* from Collection J and one *Salamandra corsica* from Collection K all showed 100% identity to the type strain, AMFP13/1, in the Netherlands and Belgium^2^.

## Discussion

We identified *Bsal*-positive urodeles in seven of 11 tested private amphibian collections across Western Europe that were epidemiologically linked to the index case in the UK^21^, including the first identification of *Bsal* in Spain. In addition to apparent subclinical infection of urodeles, both clinical disease and fatal chytridiomycosis were diagnosed in several collections. None of the anurans tested were positive for this pathogen despite being in a *Bsal*-positive collection with an absence of biosecurity, such as shared tools, water, plants and other matter being transferred between enclosures. Whilst the tested collections only form a small proportion of the number of private amphibian collections in Europe, a high percentage of those tested were found to be *Bsal*-positive. It is likely, therefore, that *Bsal* is widespread within the European private amphibian trade. This is in contrast to the situation in the United States of America, where surveys have not identified *Bsal* either in the private amphibian trade^24^, or in the wild^25,26^.

Fatal *Bsal* chytridiomycosis was confirmed using histological examination and qPCR in nine dead urodeles from three collections: one in the UK and two in the Netherlands. These deaths and diagnoses all occurred during multiple mortality events affecting a range of urodele species native to Europe, North America and the Middle East. There was a wide range of infection intensities observed in animals that died with confirmed *Bsal* chytridiomycosis (523.20–115,400.00 GE swab^−1^) (Table 2). Further research into the pathogenesis of *Bsal* will help to identify possible links between infection intensity and the likelihood of mortality, and whether any link between infection intensity and mortality is species- or life-history-specific.

The spectrum of species we found to be affected by fatal *Bsal* infection in private collections largely corroborates results of experimental infection trials^6^. Here, however, we also show mass mortality in aquatic newts, with complete eradication of the breeding groups, whereas wild urodele declines due to *Bsal* chytridiomycosis have so far only been detected in terrestrial *Salamandra salamandra*^2,3^. Although the situation in captivity might not be representative of that in the wild, it clearly shows that *Bsal* transmission and disease in newts during the aquatic life stage is possible. Also, this study expands the spectrum of susceptible host species (Supplementary Table S1), with the inclusion of the genera *Ambystoma* (*A. mexicanum*) and *Ommatotriton* (*O. ophryticus*) and further species in the genera *Neurergus* (*N. strauchii*), *Triturus* (*T. dobrogicus, T. ivanbureschi, T. karelinii, T. marmoratus*), *Salamandra* (*S. atra*) and *Lissotriton* (*L. boscai*). These additions to the list of species susceptible to *Bsal* infection confirm the potential threat of *Bsal* to western Palearctic and Nearctic urodele diversity.

It is thought that *Bsal* initially reached Europe via the import of Asian urodeles and that these species are key in the movement of the pathogen^6^. Our study shows that the movement of non-Asian urodeles can also present a biosecurity risk to captive amphibian collections. We failed to detect an association between the presence of Asian urodeles and the presence of *Bsal* or unusually high mortality in collections. The movement of non-Asian urodeles appears to have introduced the pathogen into at least three of the *Bsal*-positive collections with higher than usual levels of mortality (Collections A, C and J; Table 1). These results, coupled with the significantly higher *Bsal* infection prevalence in non-Asian vs Asian urodeles in Collections A-J, indicate that non-Asian urodeles should be considered potential vectors of *Bsal* in the private amphibian trade.

Following the emergence of *Bsal* in wild amphibians in Western Europe, there is a danger that this pathogen will cause a second amphibian chytridiomycosis panzootic, similar to the *Bd* panzootic but with a greater impact on urodeles. Once established in the wild, the disease is likely to be extremely difficult to eradicate or otherwise mitigate^27^ if its epidemiology is similar to that of *Bd*^28^. *Bd* is now established in wild amphibian populations on a global scale^29,30^ and, despite best efforts, strategies to mitigate the effect of *Bd* in the wild have only had small-scale, short-term success^31–33^. Preventing such an outcome with *Bsal* is a high priority, but the best methods by which to do this vary depending on location and are still being debated^34–36^. One area of agreement is that reducing the risk of spread to, and establishment in, wild populations is a primary objective^28,30,36–38^. The amphibian trade has been identified as the likeliest route of *Bsal* introduction to the wild in Europe^6^. We identify the widespread presence of *Bsal* in captive amphibian collections, providing further evidence that the amphibian trade may spread this pathogen both within countries and internationally. Thus, both the commercial and the private trade in amphibians need to be considered when developing measures to mitigate the spread of *Bsal*. In addition to posing a threat to the health and welfare of captive urodeles, the presence of *Bsal* in amphibian collections increases the risk of *Bsal* infection transmitting to nearby wild populations, for example via contaminated wastewater or released or escaped animals, in a similar manner to *Bd*^16^.

Further work is required to reduce the disease risks presented by the amphibian trade to wild amphibian health and conservation. This includes engagement of policy makers, conservationists and veterinarians with the commercial amphibian trade and hobbyists to develop and promote appropriate biosecurity protocols in a collaborative manner^34–36^. Such an engagement was conducted in the UK by conservation charities, government and the amphibian trade following the discovery of *Bsal* in captive amphibians^21^. Although it was well-received by the target sectors, the degree and duration of uptake was not quantified^39^. The European Food Safety Authority identified that screening captive collections, treating *Bsal* positive individuals^40,41^ and engaging with collectors to improve sanitary protocols are likely to be the most effective and feasible measures to protect captive urodeles from *Bsal*^42^.

Increasing the general levels of hygiene and biosecurity in captive collections and the amphibian trade has the additional benefits of increasing the health, welfare and longevity of captive animals while also minimising disease threats to native wild animals^43^. Such sanitary measures include the disinfection of equipment after each use^44^, the appropriate disinfection and disposal of waste and dead animals^42^, and the quarantine and pathogen screening^45^ of new arrivals before being placed into a collection. The development of best-practice sanitary protocols to be shared with the private and commercial trade is considered an important future step in *Bsal* mitigation^42^.

The management of wildlife diseases and mitigating their potential impacts on wild populations is a complex issue. The critical control point is the prevention of pathogen introduction^28,30,36–38^, although historically, international trade controls have not been implemented in response to diseases that solely affect wildlife^46^. This appears to be changing, with the United States of America, Canada and Switzerland all recently banning the importation of urodeles, citing the threat of *Bsal* to native amphibian biodiversity^47^. Also, the European Union (EU) recently announced regulation of the movement of captive urodeles in response to *Bsal*^48^. Due to concerns that blanket bans could lead to an increase in unregulated “black market” trade^42,49,50^, the EU regulations focus on developing a clean trade in *Bsal*-free animals. This decision affects both the import into the EU and the intra-EU movement of urodeles across national borders, with veterinarians required to certify that urodeles have been examined, and are negative, for clinical signs of *Bsal* infection, that they come from a population where no *Bsal* chytridiomycosis has been observed and that the urodeles have undergone mandatory pre-import pathogen screening and quarantine, with treatment of infected amphibians where required^48^.

In addition to these regulations, the development and dissemination of biosecurity guidance for the owners of captive amphibians is required to increase awareness of the risks of *Bsal* to native amphibian biodiversity and to prevent its spread into the wild. In parallel, surveillance for *Bsal* infection and disease in wild amphibians should be increased across Europe in order to identify and eradicate any incursions into the wild as quickly as possible, and before the infection can become established.

## Methods

### Ethics

All procedures performed in this study involving animals were in accordance with the laws of the UK and with the institutional guidelines of the Zoological Society of London (ZSL). Informed consent was obtained from the owner of each amphibian collection tested. This consent was given under the condition that each collection would remain anonymous. No charge was made for visiting any collection or for testing any animals included in this study for *Bsal* infection. Ethical approval for this study was provided by the ZSL Ethics Committee (ref. WAB17).

### Private collection identification

The index case of *Bsal* infection in the UK occurred in recently-purchased animals under quarantine in a zoological collection, as previously reported^21^. The animals had been purchased from a private hobbyist in the UK, from here on termed Collection A. We used contact-tracing methods to identify other private collections that could be contaminated with *Bsal*. For each private collection that had *Bsal* positive animals, we identified the source of all the animals currently held at the collection using the collector’s records. We determined that collections were epidemiologically linked if there had been movement of any animals between collections in the previous two years, either through the sale, purchase or swapping of urodeles. Epidemiologically linked collections were then approached to test their urodeles, and the process repeated. In total, during the course of this study, 16 epidemiologically linked collections were identified and approached, 11 of which granted access.

Samples were obtained between 11^th^ March 2015 and 26^th^ February 2016 by skin-swabbing urodeles at 11 private amphibian collections across Western Europe; five in the United Kingdom, three in the Netherlands, two in Spain, and one in Germany. At all collections, all non-larval (i.e. neotenic, juvenile and adult) urodeles of all species were tested, as current evidence indicates that larval urodeles are not able to be infected with *Bsal*^51^. At Collection A, all anurans were also tested. At the time of this study, it was thought that anurans could not be infected with *Bsal*^6^. Anuran amphibians were tested in Collection A as this *Bsal*-positive collection held a large number of both urodeles and anurans, with opportunity for cross-taxon transmission. As no anurans in this collection were *Bsal*-positive, and as sampling anurans added time and cost burdens, including to collection owners and could thus impact compliance, only urodeles were tested in subsequent collections visited.

Access to the collections, samples and records were obtained with the full knowledge and permission of the owner of each collection, which was given on the grounds on anonymity. Results were provided to the owners, so they could act on this information and seek veterinary advice, if required. In addition, we requested that carcasses of any amphibians that died during the course of this study were made available for pathological examination.

During each visit, each collection owner was asked how they sold/bought/exchanged animals, their rationale for doing so and the numbers of animals involved. This was done in an informal, conversational way to maximise the likelihood of obtaining the required information.

### Skin-swabbing

Skin swab samples were taken from all non-larval urodeles in each collection visited, whether they were live or dead, using a dry rayon-tipped swab (MW100, Medical Wire & Equipment, UK). We followed a well-established protocol of comprehensively swabbing the underside of the legs, feet (including between digits) and ventral surface (five swab strokes over each area) before replacing the swab into its plastic sleeve^52^. One swab was used per animal apart from in Collection K, where – at the owner’s request – five animals from within the same enclosure were sampled with each swab. Animals for which swabs were pooled always coinhabited the same enclosure. During swabbing, animals were visually examined for the presence of gross lesions and were observed for signs of *Bsal* chytridiomycosis, such as cachexia, lethargy or ataxia^2^.

### Quantitative PCR

DNA was extracted in 60 µl PrepMan Ultra following the manufacturer’s guidelines (Applied Biosystems, Foster City, CA). Samples were analysed for the presence of *Bd* and *Bsal* DNA using a duplex quantitative polymerase chain reaction (qPCR), targeting the ITS1 rRNA gene of *Bd* and 5.8S rRNA gene of *Bsal*, as described by Blooi *et al*.^45^. Each sample was run in duplicate; where a sample produced contradictory results in the duplicates, the qPCR was repeated until the duplicates gave a consistent result. Positive controls, comprising known quantities of both *Bd* and *Bsal* DNA (100, 10, 1 and 0.1 GE – zoospore genomic equivalents) and a negative control were included in each qPCR run. Infection intensity results from qPCR plates where the R^2^ value of the standard curves generated by the positive controls was less than 0.9 were determined to be inaccurate and were re-run. Infection intensities, measured in zoospore genomic equivalents per swab (GE swab^−1^), were calculated by multiplying the mean quantity output of *Bsal* positive qPCRs by 120, to account for the dilution between swab and PCR, as described by Hudson *et al*.^31^.

### Pathological investigations and diagnosis

Two sick urodeles submitted by collectors were euthanased with an overdose of the anaesthetic tricaine methanesulphonate (MS222) followed by destruction of the brain and spinal cord by pithing.

Urodele carcasses were examined for the presence of macroscopic skin lesions consistent with *Bd* or *Bsal* chytridiomycosis. Full necropsies were conducted following published guidelines^53^. Tissue samples, including forelimb, hindlimb, tail, 1 cm^2^ patch of dorsal skin and 1 cm^2^ patch of ventral skin, were taken for histological examination. These samples were fixed in neutral buffered 10% formalin, embedded in paraffin and sectioned, before being stained with haematoxylin and eosin using routine methods^54^.

For this study, animals which tested positive for *Bsal* on qPCR, whether or not gross or microscopic lesions were observed, are termed ‘*Bsal*-positive’. Following the case definition and diagnostic criteria for *Bsal* chytridiomycosis^23^, this disease was confirmed in dead animals when lesions consistent with *Bsal* infection were seen on histopathological examination and positive *Bsal* qPCR results were obtained.

### Genotyping

A subset of DNA extracts that were positive for *Bsal* on qPCR were further analysed using standard PCR with the STerF and STerR primers to target the ITS1–5.8S-ITS2 region of the *Bsal* 5.8 S rRNA gene^2^. PCR amplicons of approximately 160 base pairs^45^ were sequenced in-house at Ghent University and compared to the type strain (GenBank accession no. KC762295)^2^. This was conducted to determine whether the strain of *Bsal* in the captive collections was the same as the strain implicated in the epizootic in the Netherlands^2^.

### Statistical analysis

*Bsal* prevalence in each collection was calculated (number of animals that tested positive divided by the total population size tested). We then used the Clopper-Pearson test (also known as an exact binomial distribution confidence interval test) to determine the 95% confidence intervals for *Bsal* prevalence in each collection. Comparisons of *Bsal* prevalence in Asian versus non-Asian urodeles was conducted using a Pearson’s Chi-squared Test. For each of these tests, Collection K was excluded from the analyses, as swabs were pooled by five animals so results for individuals were not available. Fisher’s exact tests were used to examine if there was an association between the presence of Asian species of urodele in a collection and either (1) the presence of Bsal or (2) the presence of unusually high levels of mortality. All statistical analyses were conducted using R version 3.4.1.^55^.

## Electronic supplementary material

Supplementary Information

## Data Availability

All materials, data and associated protocols have been made available in the manuscript and supplementary information. We sampled animals under the condition that each collection/owner would be anonymous. We are, therefore, unable to provide details of the species and numbers sampled for individual collections as doing so could identify the collections sampled. This will not affect the ability to replicate this work: information about the number of each species sampled has been provided pooled across all collections.
