# Supplementary material for: Epidemiological tracing of Batrachochytrium salamandrivorans identifies widespread infection and associated mortalities in private amphibian collections
Source: Sci Rep. 2018 Sep 14;8:13845. doi: 10.1038/s41598-018-31800-z (PMC6138723; doi:10.1038/s41598-018-31800-z)
Supplement: Supplementary file 1 — Supplementary Information [file 41598_2018_31800_MOESM1_ESM.pdf]

Epidemiological tracing of *Batrachochytrium salamandrivorans* identifies widespread infection and associated mortalities in private amphibian collections

Supplementary information

Liam D. Fitzpatrick[1], Frank Pasmans[2], An Martel[2], Andrew A. Cunningham[1]*

[1]Institute of Zoology, Zoological Society of London, Regent's Park, London NW1 4RY, UK;

[2]Department of Pathology, Bacteriology and Avian Diseases, Faculty of Veterinary Medicine, Ghent University, Salisburylaan 133, 9820 Merelbeke, Belgium

[*] Corresponding author – a.cunningham@ioz.ac.uk; +44 (0)20 7449 6674

Supplementary information

Table S1. Total numbers of urodeles species tested for *Batrachochytrium salamandrivorans* (*Bsal*) at epidemiologically-linked Collections A-K.

**Species**	**Number of individuals**	**Number of *Bsal* positive on qPCR**	**Biogeographic region of species**
Ambystoma mexicanum	23	1	Nearctic
Calotriton asper	1	0	Western Palearctic
Chioglossa lusitanica	9	0	Western Palearctic
Cynops cyanurus	5	0	Asia
Cynops ensicauda	6	0	Asia
Cynops orientalis	5	0	Asia
Cynops pyrrhogaster	2	0	Asia
Echinotriton andersoni	2	0	Asia
Hynobius dunni	2	0	Asia
Ichthyosaura alpestris	70	9	Western Palearctic
Laotriton laoensis	5	0	Asia
Lissotriton boscai	15	4	Western Palearctic
Lissotriton helveticus	15	9	Western Palearctic
Lissotriton italicus	18	0	Western Palearctic
Lissotriton montandoni	10	0	Western Palearctic
Lissotriton vulgaris	11	0	Western Palearctic
Mertensiella caucasica	3	0	Western Palearctic
Neurergus kaiseri	13	0	Western Palearctic
Neurergus strauchii	18	1	Western Palearctic
Notophthalmus viridescens	11	8*	Nearctic
Ommatotriton ophryticus	13	5*	Asia
Ommatotriton vittatus	15	0	Asia
Plethodon glutinosus	2	0	Nearctic
Pleurodeles nebulosus	2	0	Western Palearctic
Pleurodeles waltl	44	0	Western Palearctic
Salamandra algira	20	0	Western Palearctic
Salamandra atra	20	3	Western Palearctic
Salamandra corsica	24	2	Western Palearctic
Salamandra infraimmaculata	63	0	Western Palearctic
Salamandra lanzai	7	0	Western Palearctic
Salamandra longirostris	13	0	Western Palearctic
Salamandra salamandra	169	7	Western Palearctic
Salamandrella keyserlingi	4	0	Western Palearctic
Salamandrina terdigitata	5	0	Western Palearctic
Salamandrina perspicillata	3	0	Western Palearctic
Speleomantes strinatii	6	0	Western Palearctic
Taricha rivularis	6	0	Nearctic
Taricha granulosa	9	0	Nearctic
Triturus carnifex	35	0	Western Palearctic
Triturus cristatus	28	5	Western Palearctic

Triturus dobrogicus	16	3	Western Palearctic
Triturus ivanbureschi	5	2	Western Palearctic
Triturus karelinii	18	5*	Western Palearctic
Triturus marmoratus	54	20*	Western Palearctic
Triturus pygmaeus	13	0	Western Palearctic
Tylototriton asperrimus	10	0	Asia
Tylototriton shanjing	5	0	Asia
Tylototriton verrucosus	18	0	Asia
Tylototriton yangi	6	0	Asia
**Total**	**877**	**84[#]**	

All those marked with * include five positive individuals from the pooled samples in Collection K

[#] Total number of *Bsal* positive animals includes 20 positive individuals from the pooled samples in Collection K

Supplementary information

Table S2. Total numbers of anuran species tested for *Batrachochytrium salamandrivorans* at Collection A.

**Species**	**Number of individuals**	**Number of *Bsal* positive on qPCR**	**Biogeographic region of species**
Alytes dickhilleni	2	0	Western Palearctic
Alytes muletensis	3	0	Western Palearctic
Bombina bombina	3	0	Western Palearctic
Bombina orientalis	3	0	Asia
Bombina variegata	6	0	Western Palearctic
Bufo bufo	1	0	Western Palearctic
Bufo spinosus	3	0	Western Palearctic
Bufotes viridis	6	0	Western Palearctic
Bufotes boulengeri	2	0	Western Palearctic
Bufotes pewzowi	3	0	Asia
Dischoglossus jeannea	2	0	Western Palearctic
Epidalea calamata	3	0	Western Palearctic
Hyla arborea	1	0	Western Palearctic
Hyla meridionalis	3	0	Western Palearctic
Pelobates cultripes	3	0	Western Palearctic
Pelobates fuscus	1	0	Western Palearctic
Pelodytes ibericus	2	0	Western Palearctic
Pelodytes punctatus	3	0	Western Palearctic
Pelophylax lessonae	2	0	Western Palearctic
Pipa parva	3	0	Neotropic
Rana macrocnemis	3	0	Western Palearctic
**Total**	**58**	**0**	
